# The Mechanism of Anti-Tumor Activity of 6-Morpholino- and 6-Amino-9-Sulfonylpurine Derivatives on Human Leukemia Cells

**DOI:** 10.3390/molecules28166136

**Published:** 2023-08-19

**Authors:** Marijana Leventić, Teuta Opačak-Bernardi, Vesna Rastija, Josipa Matić, Dijana Pavlović Saftić, Željka Ban, Biserka Žinić, Ljubica Glavaš-Obrovac

**Affiliations:** 1 Department of Medicinal Chemistry, Biochemistry and Laboratory Medicine, Faculty of Medicine Osijek, Josip Juraj Strossmayer University of Osijek, Huttlerova 4, 31000 Osijek, Croatia; marjukic@mefos.hr (M.L.); tbernardi@mefos.hr (T.O.-B.); 2 Department of Agroecology and Environmental Protection, Faculty of Agrobiotechnical Sciences Osijek, Josip Juraj Strossmayer University of Osijek, 31000 Osijek, Croatia; vrastija@fazos.hr; 3 Laboratory for Biomolecular Interactions and Spectroscopy, Division of Organic Chemistry and Biochemistry, Ruđer Bošković Institute, Bijenička Cesta 54, 10000 Zagreb, Croatia; jmatic@irb.hr (J.M.); dsaftic@irb.hr (D.P.S.); zeljka.ban@labena.hr (Ž.B.); bzinic@irb.hr (B.Ž.)

**Keywords:** *N*-9-sulfonylpurines, leukemia cells, anti-tumor, apoptosis, gene expression, miRNA expression, ADMET properties

## Abstract

The aim of this study was to explore the mechanism of antitumor effect of (*E*)-6-morpholino-9-(styrylsulfonyl)-9*H*-purine (6-Morpholino-SPD) and (*E*)-6-amino-9-(styrylsulfonyl)-9*H*-purine (6-Amino-SPD). The effects on apoptosis induction, mitochondrial potential, and accumulation of ROS in treated K562 cells were determined by flow cytometry. The RT-PCR method was used to measure the expression of *Akt*, *CA IX*, *caspase 3*, and *cytochrome c* genes, as well as selected miRNAs. Western blot analysis was used to determine the expression of Akt, cytochrome c, and caspase 3. The results demonstrate the potential of the tested derivatives as effective antitumor agents with apoptotic-inducing properties. In leukemic cells treated with 6-Amino-SPD, increased expression of *caspase 3* and *cytochrome c* genes was observed, indicating involvement of the intrinsic mitochondrial pathway in the induction of apoptosis. Conversely, leukemic cells treated with 6-Morpholino-SPD showed reduced expression of these genes. The observed downregulation of miR-21 by 6-Morpholino-SPD may contribute to the induction of apoptosis and disruption of mitochondrial function. In addition, both derivatives exhibited increased expression of *Akt* and *CA IX* genes, suggesting activation of the Akt/HIF pathway. However, the exact mechanism and its relations to the observed overexpression of miR-210 need further investigation. The acceptable absorption and distribution properties predicted by ADMET analysis suggest favorable pharmacokinetic properties for these derivatives.

## 1. Introduction

In cancer research, the structure similar to natural molecules provides an interesting framework for drug development. Nitrogen heterocycles, such as purine nucleobases, are the most diverse structures used by chemists for the synthesis of drugs with a wide range of biological and pharmacological activities [1]. The combination of the purine or pyrimidine backbone with sulfonamides is an attractive and versatile platform for the exploration and development of a new class of bioactive molecules [2]. Sulfonamide derivatives exhibit antitumor activity reflected in various mechanisms of action, such as alteration of cell cycle distribution, modification of mitochondrial activity, inhibition of angiogenesis, and tubulin polymerization [3,4]. In addition, numerous studies have shown that the mechanism of antitumor action of purine- and sulfonamide-based derivatives is often related to the PI3K/Akt/mTOR pathway, which is abnormally activated in many tumors and plays a key role in regulating various cellular functions, including cell size/growth, proliferation, survival, glucose metabolism, genome stability, transcription, and protein synthesis [5,6].

Apoptosis of cancer cells is a desirable outcome of chemotherapeutic treatment. Mitochondria play a critical role in regulating the initiation and execution of apoptosis. The intrinsic apoptotic pathway ultimately leads to permeabilization of the outer mitochondrial membrane and cell dissolution [7]. It is generally accepted that apoptosis triggered by various stimuli involves early mitochondrial activation, which subsequently disrupts mitochondrial functions, leads to loss of mitochondrial membrane potential, permeabilization of the outer mitochondrial membrane and release of cytochrome c, and finally causes cell death [8]. The successful use of chemotherapy in the treatment of tumors can be attributed, at least in part, to the high degree of mitochondrial priming observed in chemosensitive tumors [9].

CA IX is overexpressed in many types of tumor tissues, which allows tumors to survive in hypoxia and create an acidic microenvironment that increases tumor resistance. Sulfonamides, sulfanilamides, sulfamates, and other sulfonamide-based molecules have been identified as the most effective inhibitors of the mitochondrial enzyme carbonic anhydrase IX (CA IX). The inhibitory effect of sulfonamides is caused by the interaction of the sulfonamide anion with the zinc ion in the active site of the enzyme. Therefore, CA IX is considered an interesting target for the development of specific antitumor agents [10,11,12].

The molecular mechanisms underlying tumor progression and cell survival are very complex. MicroRNAs (miRNAs) have been found to play an important role in regulating cellular processes such as proliferation, apoptosis, tumor progression, pathogenesis, and drug resistance [13,14]. They regulate gene expression by binding to the 3′ untranslated region (UTR) of target messenger RNAs (mRNAs), resulting in inhibition of mRNA translation or degradation [15]. Some miRNAs act as tumor suppressors by targeting oncogenes, while others act as oncomirs and promote tumorigenesis by targeting tumor suppressors [16,17]. 

Evaluation of pharmacokinetic and toxic properties of the lead compound is necessary during drug development to avoid undesirable side effects. In silico ADMET assessment models (absorption, distribution, metabolism, excretion, and toxicity) can be used to calculate ADMET parameters [18].

**Figure 1:**
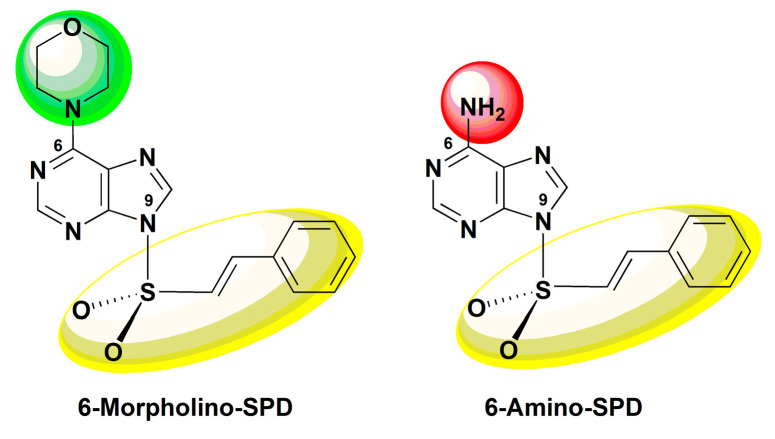
Structures of biologically active (*E*)-6-morpholino-9-(styrylsulfonyl)-9*H*-purine (6-Morpholino-SPD) and (*E*)-6-amino-9-(styrylsulfonyl)-9*H*-purine (6-Amino-SPD).

Based on the knowledge of the synthetic and biological properties of modified nucleobase scaffolds, we recently synthesized purine and pyrimidine derivatives with a sulfonamide pharmacophore attached to the nucleobase and evaluated their antitumor potential by biological studies [19,20,21,22,23]. (*E*)-6-morpholino-9-(styrylsulfonyl)-9*H*-purine and (*E*)-6-amino-9-(styrylsulfonyl)-9*H*-purine (see Figure 1) were synthesized by the conversion of new 6-chloro-9-sulfonylpurine derivatives into their corresponding 6-morpholino derivatives or by sulfonylation of adenine and 6-morpholino purine using sulfonyl chlorides. The results of the antiproliferative activity of 6-morpholino/amino-9-sulfonylpurine showed that 6-morpholino/amino purines with a *trans*-*β*-styrenesulfonyl group attached to the N9 position of purine exhibited the highest antiproliferative activity against lymphoma and leukemia cells, as well as accumulation of leukemia cells in the subG0 phase of the cell cycle, indicating apoptosis as a mode of cell death [19].

In this study, we focus on the mechanism of action of (*E*)-6-morpholino-9-(styrylsulfonyl)-9*H*-purine (6-Morpholino-SPD) and (*E*)-6-amino-9-(styrylsulfonyl)-9*H*-purine (6-Amino-SPD) on human leukemia cells. We investigate the mechanism of apoptosis induction, the effects on intracellular stress, and the changes in miRNA expression. In addition, we perform an in silico ADMET.

## 2. Results

### 2.1. Effects of 6-Amino-SPD and 6-Morpholino-SPD on Apoptosis Induction and on Mitochondrial Membrane Potential (ΔΨm) in Leukemia Cells

**Figure 2:**
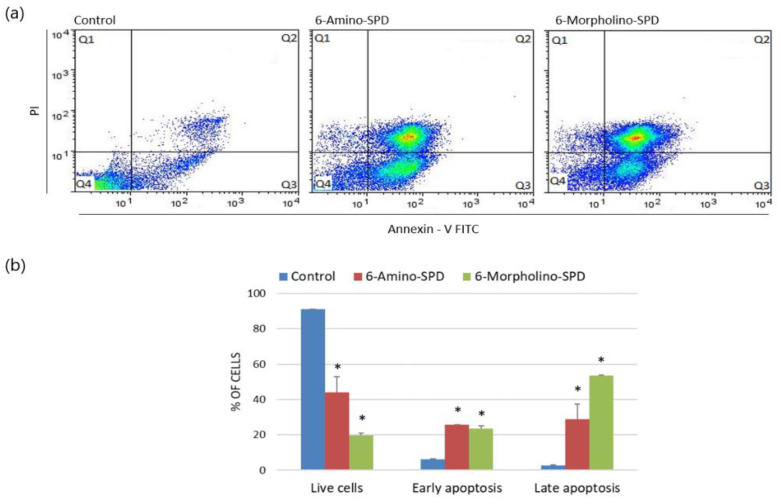
Induction of apoptosis in K562 cells treated with 6-Amino-SPD and 6-Morpholino-SPD at a concentration of 5 μM for 24 h. Data plots of control and treated cells (**a**) are divided into quadrants. Cells in Q1 are dead cells, Q2 cells are in late apoptosis, Q3 cells are in early apoptosis, and Q4 cells are live cells. The distribution of cells in each quadrant is shown graphically (**b**) in columns formed by the average percentage with standard deviations. (*) indicates a statistically significant difference compared with control (*p* < 0.05).

Annexin V-FITC fluorescence assay results show that both 6-Amino-SPD and 6-Morpholino-SPD derivatives induce apoptosis in leukemia cells. The accumulation of cells in the positive quadrant of annexin V (Q3) and propidium iodide (Q2) indicates the appearance of cells in early and late stages of apoptosis. Specifically, 6-Amino-SPD promotes a statistically significant increase in early apoptosis in 25.8% of cells and late apoptosis in 29% of cells. On the other hand, 6-Morpholino-SPD leads to a statistically significant accumulation of more than half of the cells in late apoptosis, with 53.4% of the cells in this stage, and 23.7% of the cells in early apoptosis (Figure 2).

**Figure 3:**
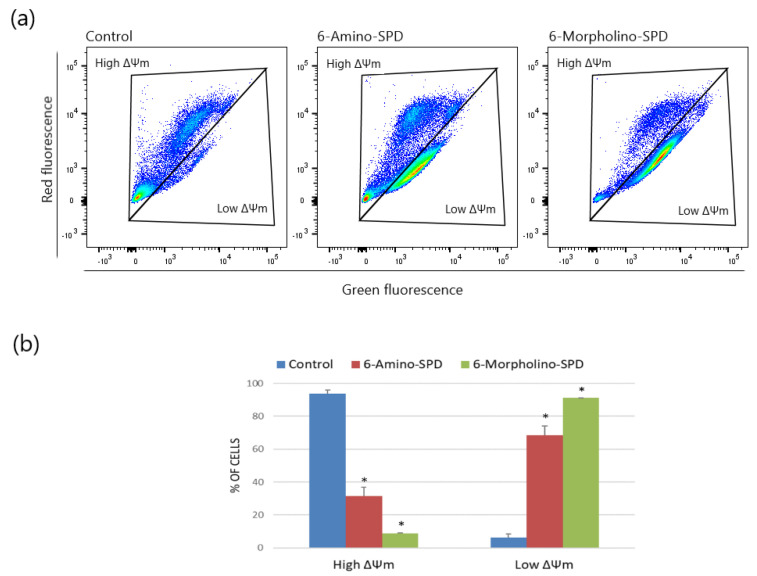
Evaluation of the change in mitochondrial membrane potential (ΔΨm) of K562 cells treated with 6-Amino-SPD and 6-Morpholino-SPD at a concentration of 5 μM for 24 h. (**a**) The changes in ∆Ψm were measured using TMRE dye. A positive control group of cells was treated with 20 μM of a protonophore carbonyl cyanide-*p*-trifluoromethoxyphenylhydrazone. (**b**) Data are expressed as mean ± standard deviation. A statistically significant *p* value is defined as *p* < 0.05 (*).

To evaluate the functional changes in mitochondria of leukemia cells treated with 6-Amino-SPD and 6-Morpholino-SPD, we measured the fluorescence intensity of the dye JC-1, which depends on the change in mitochondrial membrane potential (ΔΨm). Healthy cells with high membrane potential accumulate JC-1 aggregates, resulting in red fluorescence, while damaged cells with low membrane potential show green fluorescence. As shown in Figure 3, the derivatives caused significant damage to the majority of cells, as indicated by low ΔΨm (6-Amino-SPD: 68.6 ± 5.44%; 6-Morpholino-SPD: 91.2 ± 0.21%). Although severe mitochondrial damage was observed in cells after treatment with both derivatives, there was no significant change in the accumulation of reactive oxygen species (ROS) in leukemic cells.

### 2.2. Effects of 6-Amino-SPD and 6-Morpholino-SPD on Cytochrome c, Caspase 3, Akt, and CA IX Genes, and microRNA (miRNA) Expression Profiles

**Figure 4:**
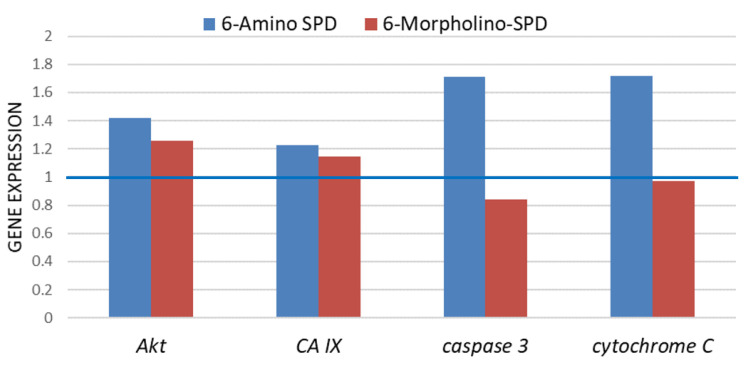
Expression of *Akt*, *CA IX*, *caspase 3*, and *cytochrome c* genes in K562 cells exposed to 6-Amino-SPD and 6-Morpholino-SPD at 2 μM in a period of 24 h. The expression results of the tested genes were normalized using the GAPDH gene. Expression in control cells is shown as a blue line.

To investigate the mechanism behind the induction of cell death in leukemic cells by 6-amino- and 6-morpholino-9-sulfonylpurine derivatives, we examined the expression of genes involved in proapoptotic signal transduction and mitochondrial function. As shown in Figure 4, cells treated with 6-Amino-SPD showed upregulation of *Akt* (by 42%), *CA IX* (by 22.8%), *caspase 3* (by 71.4%), and *cytochrome c* (by 71.8%) genes compared with untreated control cells. In contrast, cells treated with 6-Morpholino-SPD showed a slight increase in the expression of *Akt* (by 25.8%) and *CA IX* (by 14.6%) genes. Compared with untreated cells, a slight decrease in the expression of *caspase 3* was observed in leukemic cells after treatment with 6-Amino-SPD.

**Figure 5:**
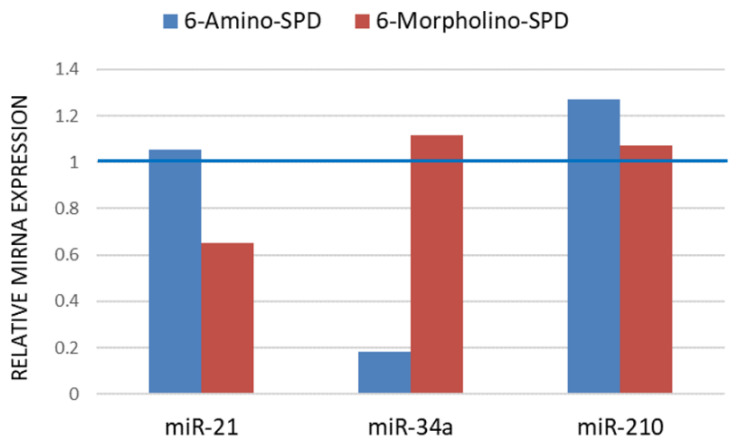
Relative miRNA expression in K562 cells after treatment with 6-Amino-SPD and 6-Morpholino-SPD compared with untreated cells (indicated by blue line). The derivatives were applied in a concentration of 2 μM for 24 h. Expression normalization was performed using miR-25.

As shown in Figure 5, the effects of the derivatives used on the expression profiles of miR-34a and miR-210 are opposite. Treatment with 6-Amino-SPD resulted in a decrease in miR-34a expression, whereas 6-Morpholino-SPD resulted in a slight increase in miR-34a expression. On the other hand, the expression of miR-210 was increased after treatment with 6-Amino-SPD, whereas no significant changes were observed after treatment with 6-Morpholino-SPD, compared with control cells. Moreover, the expression of miR-21 in the leukemic cells remained unchanged after treatment with 6-Amino-SPD, in contrast to 6-Morpholino-SPD, which caused a decrease in expression. Overall, the tested derivatives showed opposite effects on the expression profiles of miR-34a, miR-210, and miR-21 (Figure 5).

### 2.3. Changes in Protein Synthesis

**Figure 6:**
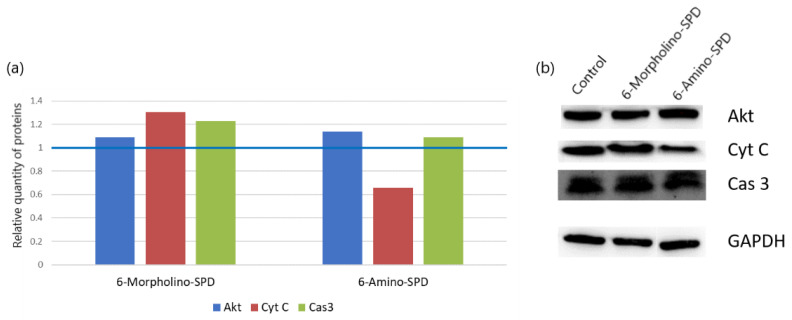
Western blot analysis of cytochrome *c* (Cyt c), caspase 3 (Cas 3), and Akt proteins in K562 cells treated with 6-Amino-SPD and 6-Morpholino-SPD at 2 μM for 24 h. Figure (**a**) shows the difference in expression after double normalization against GAPDH and control (expression of control shown as blue line). (**b**) Protein bands were separated by SDS-gel electrophoresis and visualized using chemiluminescent Immobilon Forte substrate. The ImageJ program was used to determine the density of the protein bands.

To determine the effect of 6-Amino-SPD and 6-Morpholino-SPD on protein levels induced in leukemia cells, we examined the levels of Akt, cytochrome *c*, and caspase 3 (Figure 6). All treated cells showed a slight increase in the levels of the tested proteins except cytochrome c whose levels were decreased after treatment with 6-Amino-SPD. 

### 2.4. ADMET Properties

**Figure 7:**
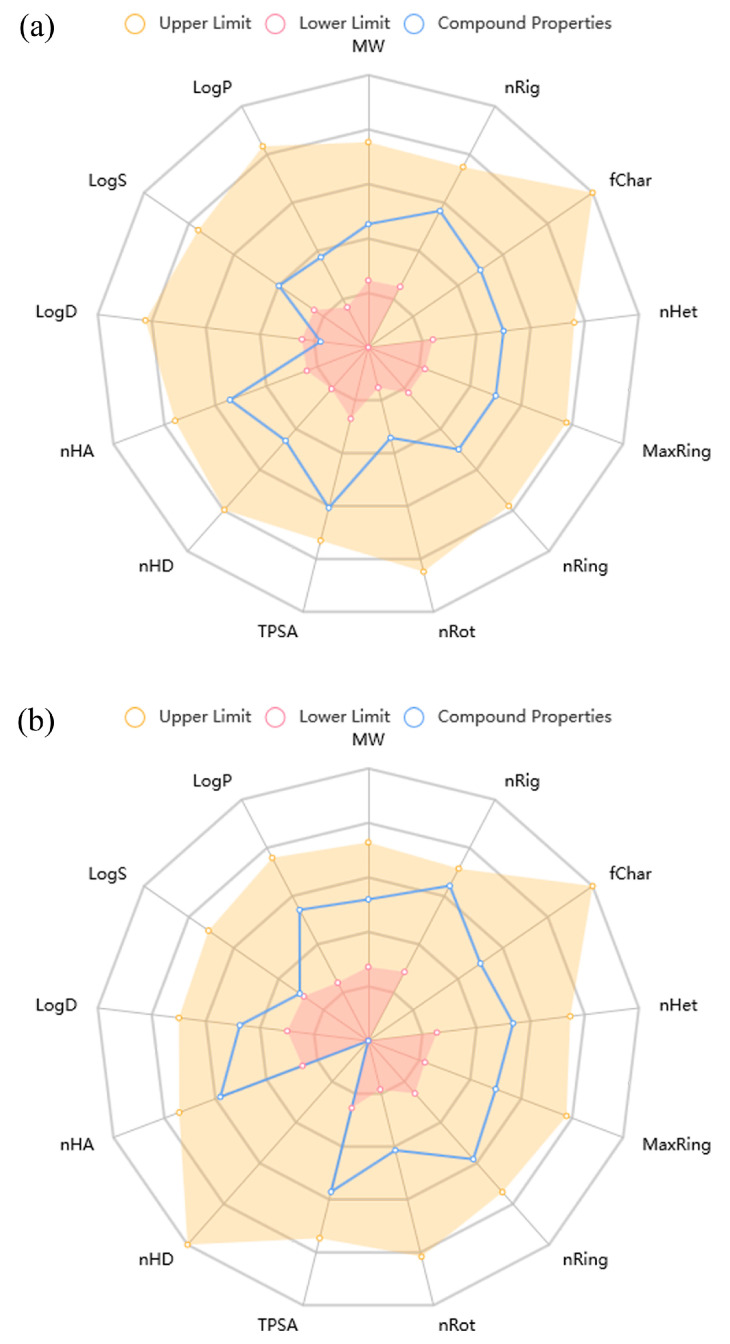
Physicochemical properties of compounds (**a**) 6-Amino-SPD and (**b**) 6-Morpholino-SPD. (MW, molecular weight; nRig, number of rings; fChar, formal charge; nHet, number of heteroatoms; MaxRing, number of atoms in the biggest ring; nRing, number of rigid bonds; nRot, number of rotatable bonds; TPSA, topological polar surface area; nHD, number of hydrogen bond donors; nHA, number of hydrogen bond acceptors; LogD, logarithm of the n-octanol/water distribution coefficients at pH = 7.4; LogS, logarithm of aqueous solubility value; LogP, logarithm of the n-octanol/water distribution coefficient).

ADMET parameters were evaluated for the two most potent compounds, 6-Amino-SPD and 6-Morpholino-SPD, against the K562 cell line. The physicochemical properties of the two compounds, known as “drug-likeness”, are shown in Figure 7. Both compounds met most of the drug-likeness properties, except that 6-Amino-SPD had a lower logD (0.761) (logarithm of the n-octanol/water distribution coefficient at pH = 7.4), which is recommended to be in the range of 1–3 [24]. 

Absorption, distribution, metabolism, excretion, and toxicity (ADMET) parameters are listed in Table 1. The compounds meet most of the medicinal chemistry properties relevant to drug-likeness, making them attractive as drug candidates according to quantitative estimates of drug-similarity (Lipinski’s rule of five) and synthetic accessibility scores. However, they do not meet the Fsp^3^ parameter, indicating a lack of sp3-hybridized carbon atoms as chiral centers. This limitation reduces the potential occupation of the target space and the efficacy of the drug. 

Both compounds exhibit excellent permeability through Caco-2 and MDCK cell membranes, which correlates with their antiproliferative activities in the same cell lines (Table 1). However, despite the good permeability, their oral bioavailability in humans (F_30%_), a crucial pharmacokinetic parameter, is estimated to be very low. The distribution of the compounds in plasma is considered very good, but there is a problem with blood–brain barrier penetration for 6-Morpholino-SPD. The poor blood–brain barrier penetration suggests that this compound may not be effective for central nervous system targets.

## 3. Discussion

Chronic myeloid leukemia (CML) is characterized by continuous activation of the BCR-ABL1 tyrosine kinase fusion gene, leading to activation of oncogenic signaling pathways. Although tyrosine kinase inhibitors are currently available for the treatment of CML, there continues to be interest in developing drugs with improved efficacy [25]. Newly synthesized derivatives of 6-chloro/morpholino/amino-9-sulfonylpurine have shown promising ability to inhibit growth in various human carcinoma, leukemia, and lymphoma cells [19]. To clarify the mechanism of action of the selected 6-Amino-SPD and 6-Morpholino-SPD derivatives and their inhibitory effects on leukemia cells, further research focused on confirming their anti-apoptotic effects. As previously mentioned, these derivatives have shown potent effect in the accumulation of leukemia cells in subG0 phase [19], indicating apoptosis characterized by fragmented DNA [26,27]. The results of this study show that both 6-Amino-SPD and 6-Morpholino-SPD derivatives induce apoptosis in the K562 cell line (Figure 2). There is an increased accumulation of cells at the late apoptosis/necrosis stage, which is particularly noticeable with 6-Morpholino-SPD. These findings are consistent with the results of previously published studies investigating compounds with similar structures. It is worth noting that indisulam, which belongs to the sulfonamide class, has anticancer activity through several mechanisms [27,28,29]. These include inhibition of carbonic anhydrase, affecting various cell cycle checkpoints, and inducing apoptosis by affecting apoptotic Bax/Bcl2 proteins. It is currently in phase I/II clinical trials [27,28,29]. In addition, purine-based compounds continue to be an attractive scaffold for the development of new antitumor agents. For example, 2,6,9-trisubstituted purine derivatives have shown proapoptotic effects on HL-60, an acute promyelocytic leukemia cell line [30].

Apoptosis is often accompanied by complicated changes in mitochondrial membrane potential that may be either an early event in the induction of apoptosis or a consequence of the apoptotic signaling pathway. These changes are reflected in the alteration of the mitochondrial transmembrane potential, ΔΨm [31]. In the treated leukemia cells, the disruption of mitochondrial potential was observed in more than 65% of the cells after 24 h of exposure, compared with the control cells (Figure 3). 

Since mitochondria are sensitive to intracellular stress, one of the activating factors in altering mitochondrial membrane function is the formation of reactive oxygen species (ROS). The formation of ROS can lead to increased and unselective mitochondrial membrane permeability and the release of proapoptotic proteins that can activate caspase-dependent or -independent pathways [32,33]. Although an increased level of ROS was expected in K562 cells treated with 6-Amino-SPD, no significant difference in the accumulation of ROS was observed compared to control cells. However, 6-Morpholino-SPD resulted in a slight increase in the levels of ROS. These results could be explained by the high expression of antioxidant proteins, such as peroxiredoxin 1/2/6 and catalase in K562 cells, as reported by Song et al. [34]. The presence of these antioxidants in K562 cells could contribute to the regulation and control of ROS levels and prevent significant changes in ROS accumulation even after treatment with 6-Amino-SPD. However, the slight increase in ROS levels observed with 6-Morpholino-SPD suggests a possible disturbance of the redox balance that could contribute to the apoptotic response observed in the cells. 

Disruption of the mitochondrial membrane can lead to increased permeability, which allows proapoptotic signaling molecules such as Bax/Bak to promote mitochondrial dysfunction and cytochrome c release [35]. Normally, cytochrome c is released from damaged mitochondria during apoptosis and migrates to the cytoplasm where it participates in the formation of the apoptosome and ultimately activates the executive caspases [36]. In the case of our study, the gene expression of *cytochrome c* was increased by 70% in the leukemic cells treated with 6-Amino-SPD (Figure 4). Interestingly, however, the protein level of cytochrome c was found to be decreased (Figure 6). We hypothesize that the decreased detection of cytochrome c is due to its rapid binding to apoptotic protease activating factor 1 to form the apoptosome. 

In contrast to 6-Amino-SPD, treatment with the 6-Morpholino-SPD derivative did not alter *cytochrome c* gene expression, but significant overexpression of cytochrome c was observed in treated cells (Figure 4 and Figure 6). Cytochrome c overexpression could enhance caspase activation and promote cell death in response to apoptotic stimuli. However, it is important to note that simple upregulation of cytochrome c alone is not sufficient to induce apoptosis [37,38].

To investigate the involvement of caspases in the activation of apoptosis in leukemia cells, the expression of caspase 3 was examined at both the gene and protein levels. After treatment with 6-Amino-SPD, an increase in the expression of *caspase 3* gene was observed in leukemia cells, indicating its activation (Figure 4). This upregulation indicates that the apoptotic signaling cascades upstream of caspase 3 were activated, resulting in increased expression of execution *caspase 3*. In addition, an increase in the protein concentration of caspase 3 was detected (Figure 6), further supporting its involvement in apoptosis induction. On the other hand, there was no significant change in the expression of *caspase 3* gene in cells treated with the 6-Morpholino-SPD derivative (Figure 4). However, overexpression of caspase 3 protein was observed (Figure 6). This suggests that caspase 3 may be activated in response to the 6-Morpholino-SPD derivative by mechanisms independent of gene expression regulation. Other factors or post-translational modifications could contribute to the observed increase in caspase 3 protein levels.

miR-21 is known to be highly expressed in various cancers, including solid tumors and hematologic malignancies such as leukemia, lymphoma, and multiple myeloma. It has been identified as an oncogene that functions primarily by inhibiting cell apoptosis [39]. Therefore, a decrease in miR-21 expression may be considered beneficial in promoting apoptosis and cell death. In the context of chronic myeloid leukemia (CML), miR-21 has been extensively studied as an important regulator of gene expression that may influence the efficacy of tyrosine kinase inhibitors (TKIs) commonly used in to treat CML [40]. Previous research by Alves et al. has shown that low miR-21 levels at the time of CML diagnosis are associated with a better response to TKI therapy, making it an attractive target for drug therapy [41]. At CML, the constitutive activity of tyrosine kinases downstream stimulates multiple signaling pathways, including PI3K/Akt and MAPK/ERK. miR-21 exerts its effect on CML cells by targeting PTEN, a protein that inhibits the PI3K/Akt signaling pathway. By downregulating PTEN, miR-21 promotes the survival of CML cells by preventing the induction of proapoptotic mitochondrial factors [39,41]. The decreased level of miR-21 in K562 cells induced by 6-Morpholino-SPD, as shown in Figure 5, is consistent with the impaired mitochondrial potential and induction of regulated cell death. On the other hand, 6-Amino-SPD showed no significant effect on miR-21 expression. This may suggest that the mechanism of action of 6-Amino-SPD in triggering cell death and regulating mitochondrial function may involve other factors or pathways that are independent of miR-21.

miR-34 is a member of the miRNA tumor suppressor family that has been identified as a target of the tumor suppressor gene p53. miR-34a plays a crucial role in suppressing tumor growth and inhibiting cancer progression by targeting multiple tumor-promoting processes, such as promoting apoptosis, autophagy, and cell cycle arrest [42]. In the K562 cell line, there is loss of one allele and an insertion mutation in exon 5 of the other allele, resulting in a frameshift mutation and expression of a truncated p53 protein [43,44]. This genetic alteration in K562 cells impairs p53 functionality. However, studies by Li et al. suggest that the modulation of miR-34a can occur through various mechanisms, including transcriptional regulation, post-transcriptional regulation, and epigenetic regulation, suggesting that the expression of miR-34a can be regulated independently of p53 [42]. In this study, it was observed that treatment with 6-Morpholino-SPD induced apoptosis in K562 cells and was associated with increased expression of miR-34a. On the other hand, treatment with 6-Amino-SPD resulted in decreased expression of miR-34a. These results suggest that the regulation of miR-34a expression may be independent of p53 in this particular context.

In addition, treatment with the derivatives, 6-Amino-SPD and 6-Morpholino-SPD, resulted in increased expression of the genes *CA IX* and *Akt*. Carbonic anhydrase IX (CA IX) is a mitochondrial enzyme that plays a role in essential cellular functions associated with tumor growth and metastasis, including pH regulation, cell survival, and adhesion/migration. The transmembrane CA IX is an interesting target for small inhibitory molecules based on sulfonamides/sulfamates because they can interact with the zinc ion in the active site of the enzyme [45]. The expression of *CA IX* is regulated by several signaling molecules, with HIF-1 being a primary regulator that can be stimulated by the PI3K/Akt pathway. Akt is a key player in the PI3K/Akt signaling pathway, and its activation may contribute to the upregulation of *CA IX* expression [46]. In this study, the increased expression of *Akt* and *CA IX* genes after treatment with the SPD derivatives suggests that they are involved in the regulation of CA IX expression, possibly through the Akt/HIF-1 pathway. 

miR-210 has been associated with the hypoxic response and regulation of the PI3K/Akt pathway. Its overexpression has been associated with poor prognosis in acute myeloid leukemia (AML). miR-210 exhibits pleiotropic effects and may regulate mitochondrial metabolism and apoptosis, among other cellular processes. It is worth noting that miRNAs can exert different effects depending on their cellular location and the specific signaling pathways they target [47,48,49]. The expression of miR-210 was not reduced in this study after treatment with the SPD derivatives (Figure 5), which is consistent with the results obtained for the gene expression of *Akt* and *CA IX*. 

In silico evaluation of the pharmacokinetic and toxic properties of the lead compound is necessary during drug development to avoid undesirable side effects. The results of ADMET parameter calculation indicate that 6-Amino-SPD and 6-Morpholino-SPD have potential as antitumor agents with satisfactory ADMET properties. The prediction of their ADMET properties indicated their satisfactory absorption and distribution properties. However, the problem could be their low excretion capacity and toxic effect. It was estimated that 6-Morpholino-SPD has a probability of 84.8% to be a substrate of CYP2C9 enzyme. The excretion of 6-Amino-SPD was found to be low and its half-life medium, as was that of 6-Morpholino-SPD. They also have a low probability of eye irritation. However, the compound 6-Amino-SPD has a high probability of acute oral toxicity in rats (63.0%) and respiratory toxicity (92.1%). When 6-Morpholino-SPD is applied as a skin product, it could cause allergic contact dermatitis (93.6%). The same compound may also cause hepatotoxicity in humans (80.6%) and a mutagenic effect closely related to carcinogenicity (AMES toxicity, 80.6%). Both compounds were predicted to have a high probability of liver injury (6-Amino-SPD 97.5%, 6-Morpholino-SPD 99.3%).

## 4. Materials and Methods

### 4.1. Cell Culturing

Chronic myeloid leukemia in blast crisis (K562) was cultured in RPMI 1640 medium (Lonza, Basel, Switzerland), supplemented with 10% FBS (Gibco, Thermo Fisher Scientific Inc., Leicestershire, UK), 2 mM glutamine, 1 mM sodium pyruvate, and 10 mM HEPES. Cells were grown in a humidified atmosphere under 37 °C/5% of CO_2_ gas in a CO_2_ incubator (IGO 150 CELLlifeTM, JOUAN, Thermo Fisher Scientific, Waltham, MA, USA). K562 cells were tested and authenticated using the short tandem repeat profiling (STR) analysis method prior to experiments.

### 4.2. Annexin V/PI Assay

Apoptosis was detected on K562 cells after application of 5 μM of 6-Amino-SPD and 6-Morpholino-SPD for 24 h, and doxorubicin at a concentration of 0.1 μM was used as a positive control. Cells were seeded in 6-well plates at a concentration of 5 × 10^5^ cells per well. On the day of analysis, cells were resuspended in 500 μL 1× buffer from the Annexin V-FITC apoptosis detection kit (Abcam, Cambridge, UK) after centrifugation at 3000 rpm/5 min. Cells were stained with 5 μL Annexin V-FITC and PI and analyzed on a flow cytometer (FascCanto II, BD Bioscience, San Jose, CA, USA) using Flowjo software version 10.7 (Flowjo LLC, Ashland, OR, USA).

### 4.3. Measurement of ΔΨm

Mitochondrial membrane potential (ΔΨm) was measured in K562 cells treated with 5 μM 6-Amino-SPD and 6-Morpholino-SPD. Cells were seeded on 6-well plates at a concentration of 5 × 10^5^ cells per well. After 24 h of incubation, the cells were collected and centrifuged at 600× *g* for 4 min at 4 °C. The supernatant was removed from the cells, and JC-1 staining solution prepared according to the manufacturer’s instructions (mitochondria staining kit for mitochondrial potential changes detection, Sigma-Aldrich, St. Louis, MO, USA) was added to the cells. At the end of the incubation, the JC-1 dye was removed from the cells by centrifugation at 600× *g*, 4 min at 4 °C, and washed with cold JC-1 buffer. Analysis was performed on a flow cytometer (FascCanto II, BD Bioscience, San Jose, CA, USA) using FlowJo software 10.7 (Flowjo LLC, Ashland, OR, USA).

### 4.4. Determination of Intracellular Reactive Oxygen Species (ROS)

K562 cells, at a concentration of 5 × 10^5^ cells/mL PBS, were incubated for 1 h in an incubator at 37 °C/5% CO_2_ with 6-Amino-SPD and 6-Morpholino-SPD (5 μM). At the end of the incubation, a reagent for detecting ROS was added to the cells according to the manufacturer’s instructions from the Fluorometric Intracellular Ros Kit (Sigma-Aldrich, St. Louis, MO, USA), and the cells were then incubated for 30 min. Cell analysis was performed on a flow cytometer (FascCanto II, BD Bioscience, San Jose, CA, USA) using FlowJo software 10.7 (Flowjo LLC, Ashland, OR, USA).

### 4.5. Real-Time Quantitative PCR (qPCR)

K562 cells were cultured with or without 2 μM 6-Amino-SPD and 6-Morpholino-SPD for 24 h. Total RNA was isolated using the RNeasy Mini kit (Qiagen, Hilden, Germany), and reverse transcription of RNA was performed using the PrimeScript™ 1st strand cDNA Synthesis Kit (Takara Bio Eu, Saint-Germain-en-Laye, France). Amplification was performed in a QuantStudio 5 Real-Time instrument (Thermo Fisher Scientific, Rockford, IL, USA). PCR was performed in 20 μL volume using the GoTaq^®^ Flexi DNA Polymerase protocol (Promega, Madison, WI, USA) for an initial denaturation of 2.5 min at 95 °C, followed by 40 cycles of 95 °C for 30 s and 1.5 min for annealing of primers (*GAPDH* at 68 °C, *caspase 3*, *cytochrome c*, *Akt1*, and *CA IX* at 57 °C). Ct values of the samples were normalized to *GDPDH*. Relative expression was calculated using the 2^−ΔΔCt^ method [24]. The following amplification primers were used: *GAPDH* 5′-CCA TCA ATG ACC CCT TCA TTG ACC-3′ sense, 5′-GAA GGC CAT GCC AGT GAG CTT CC-3 antisense; *Caspase 3* 5′-TCG GTC TGG TAC AGA TGT CG-3′ sense, 5′-CAT ACA AGA AGT CGG CCT CC-3′ antisense; *Cytochrome C* 5′-AGA ACA AAG GCA TCA TCT GGG-3′ sense, 5′-TCA GCT GTA GCC GAG AGT CA-3′ antisense; *Akt1* 5′-CTT CCT CAC AGC CCT GAA GT-3′ sense, 5′-TAA TGT GCC CGT CCT TGT CC-3′ antisense; and *CA IX* 5′-TGA GGA AGG CTC AGA GAC TCA-3′ sense; 5′-TCA GCT GTA GCC GAG AGT CA-3′ antisense. 

### 4.6. miRNA Expression

The total RNA is transcribed according to the manufacturer’s instructions using the TaqMan Advanced miRNA cDNA Synthesis Kit (Thermo Fisher Scientific, Rockford, IL, USA). The miRNA sequences hsa-miR-25-3p and hsa-miR-93-5p [50] were used as controls, and hsa-miR-21-5p, hsa-miR-34a-5p, and hsa-miR-210-3 were used as targets. TaqMan assay for miRNA analysis was performed using the QuantStudio 5 real-time instrument (Thermo Fisher Scientific, Rockford, IL, USA). The fold difference in miRNA expression was determined using the 2^−ΔΔCt^ method described by Livak et al. [51] and normalized to control miRNA. 

### 4.7. Western Blot

Cultured K562 cells were treated with 2 μM 6-Amino-SPD and 6-Morpholino-SPD for 24 h. After treatment, the cells were lysed and the cell lysate was electrophoresed on the Mini Protean System (BIO-RAD, Hercules, CA, USA). After electrophoresis on a precast gradient of 4–15%, gel proteins were transferred to a PVDF membrane. The membrane was blocked with 3% BSA in TBST for 1 h before binding the primary antibody overnight at 4 °C. Proteins were identified using an HRP-conjugated secondary antibody (Cell Signaling Technology, Danvers, MA, USA) and Immobilon Forte chemiluminescent substrate (Milipore, MA, USA). Images were acquired using the ChemiDoc Imaging System (BIO-RAD, Hercules, CA, USA) and quantified using ImageJ software 1.53k Java1.8.0_172. Quantified data were normalized against GAPDH and untreated cells.

### 4.8. ADMET Properties Prediction

The absorption, distribution, metabolism, excretion, and toxicity (ADMET) parameters of compounds were evaluated using ADMETlab 2.0. The ADMETlab 2.0 server is freely available at https://admetmesh.scbdd.com/ (accessed on 2 February 2023).

### 4.9. Statistical Analysis

Where appropriate, data were analyzed using one-way analysis of variance (ANOVA) with Bonferroni corrections to examine statistical differences between the treatment groups and the untreated control. The analysis was performed using the XLSTAT add-on for Excel.

## 5. Conclusions

The results of this study demonstrate the potential of 6-Morpholino-SPD and 6-Amino-SPD as potent antitumor agents with apoptotic-inducing properties. The observed impairment of mitochondrial potential and increased expression of *caspase 3* and *cytochrome c* genes in leukemic cells treated with 6-Amino-SPD suggest that they induce apoptosis via the intrinsic mitochondrial pathway. The decreased expression of these genes in leukemic cells treated with 6-Morpholino-SPD suggests that this derivative may induce apoptosis via a different mechanism. 

Down-regulation of miR-21 by 6-Morpholino-SPD could contribute to the induction of apoptosis and disruption of mitochondrial function in K562 cells. Modulation of miR-21 expression, as observed with the 6-Morpholino-SPD derivative, may represent a strategy to promote cell death and improve the efficacy of treatment of CML. The increased expression of *Akt* and *CA IX* genes suggests that 6-Amino-SPD and 6-Morpholino-SPD may activate signaling pathways involved in cell survival and tumor progression. Taken together, these results suggest that 6-Amino-SPD and 6-Morpholino-SPD can induce apoptosis in leukemic cells through different signaling pathways. In addition, our results suggest that the regulation of CA IX expression by the tested derivatives may occur through the Akt/HIF pathway, but the exact mechanism and its relationship with miR-210 need further investigation. 

The acceptable absorption and distribution properties predicted by ADMET analysis suggest favorable pharmacokinetic properties for these derivatives.

In conclusion, this study provides valuable insights into the apoptotic-inducing effects, molecular mechanisms, and pharmacological properties of the tested derivatives, paving the way for further research and potential development of these compounds as effective treatments for cancer.

## Figures and Tables

**Table 1:** Absorption, distribution, metabolism, excretion, and toxicity (ADMET) parameters of compounds 6-Amino-SPD and 6-Morpholino-SPD.

	Amino-SPD	Morpholino-SPD	Results Interpretation
Medicinal Chemistry			
QED	0.885	0.687	>0.67 attractive compounds; ≤0.67 poor
Synthetic accessibility score	3.736	2.709	≤6 excellent; >6 poor
Fsp^3^	0.077	0.235	≥0.42 excellent; <0.42 poor
Lipinski rule	Accepted	Accepted	
Absorption			
Caco-2 permeability	−5.009	−4.479	>−5.15 excellent; <−5.15 poor
MDCK permeability	6.5 × 10^−6^	1.70 × 10^−5^	>2 × 10^−6^ cm/s excellent; <2 × 10^−6^ cm/s poor
F_30%_	0.996	0.813	0–0.3 excellent; 0.3–0.7 medium; 0.7–1.0 poor
Distribution			
Plasma protein binding	17.01%	46.09%	≤90% excellent; >90% poor
Volume distribution	1.121	0.812	0.04–20 excellent; otherwise poor
BBB penetration	0.359	0.923	0–0.3 excellent; 0.3–0.7 medium; 0.7–1.0 poor
Fraction unbound in plasma (Fu)	82.10%	61.47%	>20% high Fu; 5–20% medium Fu; <5% low Fu
Metabolism			
CYP2C9 substrate	0.636	0.848	probability of being substrate 0–1
Excretion			
Clearance of a drug	4.683	6.78	≥5 excellent; <5 poor
Half-life of a drug (T_1/2_)	0.657	0.456	0–0.3 excellent; 0.3–0.7 medium; 0.7–1.0 poor
Toxicity			
hERG blockers	0.037	0.025	0–0.3 non-toxic; 0.3–0.7 medium; 0.7–1.0 toxic
Human hepatotoxicity	0.488	0.806	probability of being toxic 0–1
Drug-induced liver injury	0.975	0.993	probability of being toxic 0–1
AMES toxicity	0.216	0.796	probability of being toxic 0–1
Rat oral acute toxicity	0.630	0.141	probability of being toxic 0–1
Skin sensitization	0.259	0.936	probability of being toxic 0–1
Carcinogenicity	0.364	0.372	probability of being toxic 0–1
Eye irritation	0.029	0.095	probability of being toxic 0–1
Respiratory toxicity	0.921	0.378	probability of being toxic 0–1

QED, quantitative estimate of drug-likeness; Fsp^3^, the number of sp3 hybridized carbons/total carbon count; Caco-2 (human colon adenocarcinoma cell line) permeability; MDCK (Madin–Darby canine kidney cells) permeability; F_30%_, human oral bioavailability 30%; BBB (blood–brain barrier) penetration; CYP2C9, cytochrome P450 family 2 subfamily C member 9; hERG (human ether-a-go-go related gene) blockers; AMES toxicity, Ames test for mutagenicity.

## Data Availability

Not applicable.
